# Demographic Parameters and Life History Traits of *Neoseiulus cucumeris* (Oudemans) (Acari: Phytoseiidae) Influenced by Different Temperatures and Two Types of Food

**DOI:** 10.3390/insects16080777

**Published:** 2025-07-29

**Authors:** Mohammed M. E. Elmoghazy, Eslam Kamal Fahmy, Tagwa Salah Ahmed Mohammed Ali, Mohamed El-Sherbiny, Rasha Hamed Al-Serwi, Moaz Abulfaraj, Dalia M. A. Elsherbini

**Affiliations:** 1 Agriculture Zoology and Nematology Department, Faculty of Agriculture, Al-Azhar University, Cairo P.O. Box 11884, Egypt; mmeelmoghazy@gmail.com; 2 Department of Physiology, College of Medicine, Northern Border University (NBU), Arar 91431, Saudi Arabia; eslam.kamal.fahmy@gmail.com; 3 Department of Physiology, College of Medicine, Zagazig University, Zagazig 44519, Egypt; 4 Department of Clinical Laboratory Sciences, College of Applied Medical Sciences, Jouf University, P.O. Box 2014, Sakaka 72388, Saudi Arabia; tsalah@ju.edu.sa; 5 Department of Parasitology and Medical Entomology, Faculty of Medical Laboratory Science, Sudan University of Science and Technology, Khartoum P.O. Box 407, Sudan; 6 Department of Basic Medical Sciences, College of Medicine, AlMaarefa University, P.O. Box 71666, Riyadh 11597, Saudi Arabia; msharbini@um.edu.sa; 7 Department of Basic Dental Sciences, College of Dentistry, Princess Nourah bint Abdulrahman University, P.O. Box 84428, Riyadh 11671, Saudi Arabia; rhalserwi@pnu.edu.sa; 8 Department of Surgery, Faculty of Medicine, King Abdulaziz University, Jeddah 21589, Saudi Arabia; mabolafaraj@kau.edu.sa; 9 Department of Anatomy, Faculty of Medicine, Mansoura University, Mansoura 35516, Egypt

**Keywords:** predaceous mite, date palm pollen, *Tetranychus urticae*, breeding, life table

## Abstract

Studying the nutritional ecology of *Neoseiulus cucumeris* (Oudemans) at different temperatures is a fundamental tool for improving mass production for use in biological control of pest mites. The current research studied the impact of both food types and temperatures on the life history and demographic parameters of the predator mite *N. cucumeris*. Mite cultures in the laboratory were developed using *Tetranychus urticae* Koch, and *N. cucumeris* was collected from field plants. The developmental stages of *N. cucumeris* fed on date palm pollen and the immature stages of *T. urticae* were investigated in a laboratory setting at different temperatures. Our research revealed that *N. cucumeris* readily accepted both food types at all the tested temperatures. The developmental stages and adult longevity of *N. cucumeris*, both female and male, decreased dramatically when the temperature increased from 18 °C to 34 °C. The net reproductive rate (*R*_0_) reached its greatest values of 22.52 and 9.72 offspring/individual at 26 °C, and the intrinsic rate of increase (*r_m_*) reached its maximum values of 0.17 and 0.13 day^−1^ at 34 °C and minimum of 0.12 and 0.10 day^−1^ at 18 °C, when fed on date palm pollen and immature stages of *T. urticae*, respectively. Conversely, the average generation time (*T*) showed a notable reduction from 22.48 to 16.48 and 20.88 to 16.76 days, accompanied by an upsurge in temperature from 18 °C to 34 °C, when fed on date palm pollen and immature stages of *T. urticae*, respectively. The finite rate of growth (λ) exhibited distinct variations, reaching its highest value at 34 °C, 26 °C, and 18 °C when fed on date palm pollen and immature stages of *T. urticae*, respectively. From these results, we can conclude that *N. cucumeris* was successfully fed date palm pollen as an alternate source of nourishment. In addition, the immature stages of *T. urticae* are suitable as food sources for *N. cucumeris* because they shorten the mean generation time. Therefore, the success of mass-rearing the predator mite *N. cucumeris* on a different, less expensive diet, such as date palm pollen, and determining the most suitable temperature for it has increased its spread as a biocontrol agent.

## Simple Summary

This study investigated the nutritional ecology of *Neoseiulus cucumeris* (Oudemans) at different temperatures, which is crucial for its conservation and improvement as a natural pest management agent. Mite cultures were developed using *Tetranychus urticae* Koch and *N. cucumeris* collected from field plants. The developmental stages of *N. cucumeris* fed on date palm pollen and the immature stages of *T. urticae* were investigated at different temperatures. These results suggest that *N. cucumeris* successfully fed on date palm pollen as an alternate source of nourishment at all tested temperatures and that the immature stages of *T. urticae* were suitable as food for *N. cucumeris* because they shortened the mean generation time for both females and males when temperatures increased from 18 °C to 34 °C. The net reproductive rate (*R*_0_) reached its greatest values at 26 °C, and the intrinsic rate of increase (*r_m_*) reached its maximum values at 34 °C and minimum at 18 °C, when fed on date palm pollen compared with immature stages of *T. urticae*. The success of mass-rearing the predator mite *N. cucumeris* on a different, less expensive diet, such as date palm pollen, and determining the most suitable temperature for it has increased its spread as a biocontrol agent.

## 1. Introduction

The Phytoseiidae family comprises predatory mites that primarily consume small insects and phytophagous mites [1]. Members of this family play a key role in the biological control of the two-spotted spider mite *Tetranychus urticae* Koch, particularly in greenhouse crops. Additionally, certain species within this family feed on microscopic soil organisms, pollen, and plant exudates [2,3,4]. This family is widely distributed worldwide, with more than 90 genera including 2800 species; few of them have been thoroughly investigated for their efficacy as biological control agents [5,6].

The polyphagous two-spotted spider mite *T. urticae* is a globally prevalent pest that poses significant threats to various horticultural crops grown in both open fields and greenhouse environments. It infests over 1100 host plant species, including more than 150 species of strategic importance [7,8,9,10,11,12]. Given the rapid development of pesticide resistance, coupled with short life cycles and high population growth rates in various cultivars, it is important to consider integrated management strategies that incorporate the use of biological control agents [13,14,15].

Since its first use for thrip biocontrol in 1985 [6], *Neoseiulus cucumeris* (Oudemans) has gained global recognition as a generalist predator [2,16,17,18]. It is well recognised for its ability to control aphids, psyllids, whiteflies, mites, and thrips [19,20,21,22,23]. The potential of *N. cucumeris* has already been evaluated on *T. urticae* as the main prey and *Tyrophagus putrescentiae* (Schrank) as a factitious prey for 30 generations [24].

Feeding on a pollen diet is a considerable characteristic of *N. cucumeris* that facilitates the cost-effective mass rearing of large numbers of this predator [24,25]. Proteins and essential amino acids are abundant in plant pollen, which provides phytoseiid mites with a high-quality diet [26,27].

Understanding the biological traits and life table parameters of phytoseiid predators is a crucial step for assessing their potential as predators [28,29,30]. Previous studies have demonstrated that many significant elements, including host plant, prey species, temperature, and relative humidity, have different impacts on the life table characteristics of phytoseiid mites [31,32,33,34].

This study enhances our understanding of the effects of varying temperatures and dietary conditions on the growth and reproductive capabilities of the predatory mite *N. cucumeris*. This knowledge is essential for optimising breeding techniques and large-scale production of predatory mites for biological control.

## 2. Materials and Methods

### 2.1. Laboratory Rearing of Tetranychus urticae

*Tetranychus urticae* was obtained from field plants (Eggplant, *Solanum melongena* L. and Okra, *Hibiscus esculentus* L., location 29.97° N 40.21° E) and used to generate mite cultures in the laboratory. *Tetranychus urticae* was maintained for five weeks on laboratory-grown bean plants (*Phaseolus vulgaris* L.) before consumption. Mite cultures were kept in the laboratory at 25 ± 2 °C, 60 ± 5% relative humidity (RH), and a photoperiod of 16:8 h (light–dark).

### 2.2. Laboratory Rearing of Predator Mites

*Neoseiulus cucumeris* was obtained from field plants (Mango, *Mangifera indica* L., location 29.97° N 40.21° E) and reared on detached mulberry leaves in the laboratory at temperatures, humidity, and photoperiods similar to those reared on *T. urticae*. Prior to use, freshly collected mulberry leaves, *Morus alba* L. [31,35,36], were cleaned with a water spray and then allowed to dry. To prevent mites from escaping, each leaf was placed on a layer of damp cotton wool in foam dishes (20 × 15 cm in length and 3 cm in depth). This arrangement ensured that the leaves remained fresh for approximately one week. Predator-rearing dishes were divided into two groups: the first group was fed date palm pollen, and the second was fed on mixed stages of *T. urticae* on a piece of bean leaves from the breeding colony [24,37]. A minimum of five to seven generations of *N. cucumeris* were produced according to this method prior to the use of the colony in this experiment.

### 2.3. Life Table Study

Six treatment groups (reared on two types of food at three different temperatures) were established. For each group, 40 fresh mulberry leaf discs, each measuring 5 cm in diameter, were placed on damp cotton inside the foam dishes. Each foam dish consisted of five discs made of mulberry leaves (eight foam dishes/group), with each disc enclosed with damp cotton to effectively prevent mites from escaping. Freshly laid eggs were individually transferred from a stock culture of *N. cucumeris* to these discs (one egg/disc). Two types of food were used to rear the predator: The first type was date palm pollen *Phoenix dactylifera* L. harvested during the flowering season from date palm orchards; the amount used was 3–5 mg/2 days/disc. The second type was the immature stages of *T. urticae*, 15–40 prey/day/disc, depending on the predator’s growth stage. The experimental units were placed in three climate chambers at temperatures of (18, 26, and 34) ± 1 °C, 60 ± 5% relative humidity, and a photoperiod of 16:8 h (light–dark), which are suitable for *N. cucumeris* breeding [38,39]. The experimental units were examined twice a day, at 12 h intervals, to assess the duration required for the survival and developmental stages of the individuals. Once the adults emerged, each female was paired with one male and transferred to a separate experimental unit (disc of mulberry leaves treated as explained previously) while maintaining the previous food and environmental conditions. Data on adult longevity, fertility, and survival were collected by daily observations until the end of the study (life span of *N. cucumeris*).

### 2.4. Data Analysis

SPSS version 26 (SPSS Inc., Chicago, IL, USA) was used for the analysis of numerical data. The Shapiro–Wilk test was utilised to assess normality (*p*-value > 0.05 showed a normal distribution), and Levene’s test was conducted to verify the homogeneity of variance. To compare means across groups regarding the mean duration of the developmental stages of *N. cucumeris*, adult female longevity, and fecundity influenced by different temperatures for each type of food, we performed one-way analysis of variance (ANOVA) followed by post hoc tests (LSD Fisher). To compare the two types of food during the life cycle of *N. cucumeris* at the same temperature, an independent-sample t-test was applied. The interaction between total immature stage, life cycle, longevity, and life span period of *N. cucumeris* at different temperatures and the type of food was analysed using repeated-measures ANOVA conducted by GraphPad Prism 8.0.2. Statistical significance was set at *p* < 0.05. Graphs were created using the GraphPad Prism 8.0.2 and Microsoft Excel applications. Values for life table parameters were calculated using Life 48 BASIC software [40].

$$R_{0}=\sum \left(l_{x}\;m_{x}\right)\;$$

$$T=\frac{\sum \left(x\;l_{x}\;m_{x}\right)}{R_{0}}$$

$$r_{m}=\sum \left(e^{-r_{m}x}\;l_{x}\;m_{x}\right)=1$$

$$\lambda =e^{r_{m}}$$

*R*_0_ = net reproductive rate.

*x* = actual female age (time from egg stage).

*l_x_* = rate of survival.

*m_x_* = female progeny per female.

*T* = mean generation time.

*r_m_* = intrinsic rate of increase.

*λ* = finite rate of increase.

## 3. Results

### 3.1. Influence of Temperature

At all tested temperatures, *N. cucumeris* successfully completed its life cycle. The mean duration of the egg and all immature stages decreased as temperature increased, with no statistically significant differences observed (*p* > 0.05) (Table 1 and Table 2). Among the immature stages, male larvae exhibited the shortest duration, followed by the protonymph and deutonymphal stages. A similar pattern was observed in females, although they demonstrated longer durations than males across all three stages. There were significant differences (*p* < 0.05) in longevity and life span between females and males at 18 °C, 26 °C, and 34 °C. The female and male life spans were the longest (46.21 and 40.80 days) at 18 °C and the shortest (31.75 and 26.60 days) at 34 °C, respectively, when fed date palm pollen. Similarly, when feeding on *T. urticae* immature stages, the female and male life spans were the longest (42.50 and 37.91 days) at 18 °C and the shortest (28.50 and 22.68 days) at 34 °C, respectively. In addition, the results indicated a significant difference (*p* < 0.001) in longevity and life span between females and males at 18, 26, and 34 °C when fed on date palm pollen and *T. urticae* immature stages (Table 1 and Table 2).

**Figure 1:**
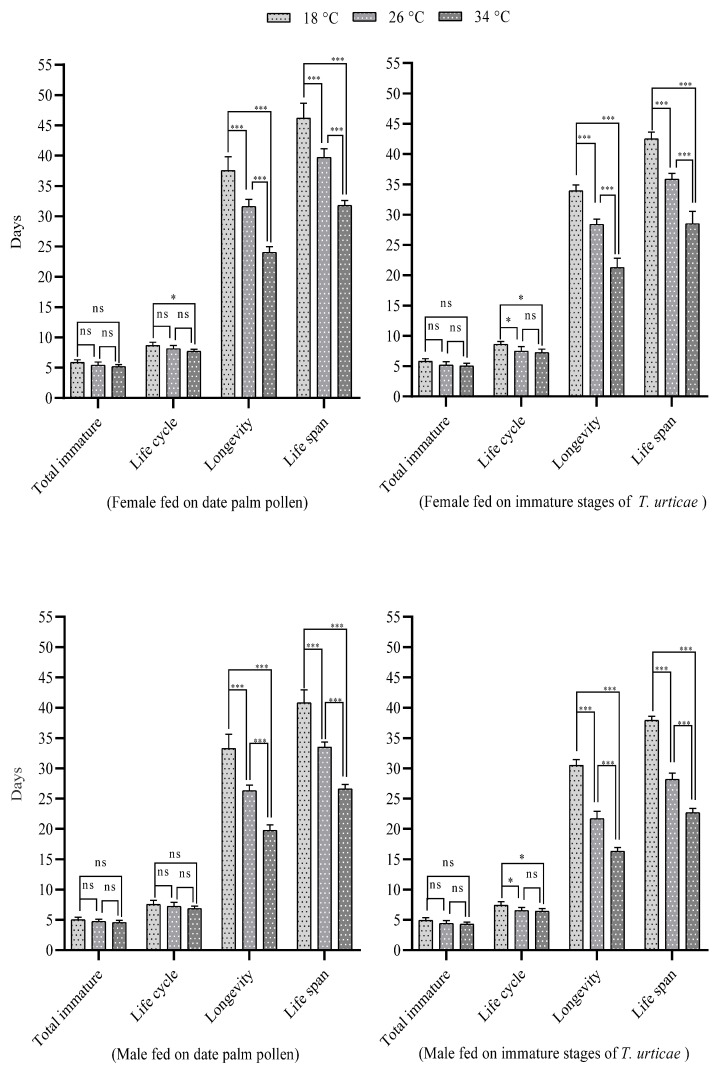
Influence of temperature on the total immature stage, life cycle, longevity, and life span period (female and male) of *N. cucumeris* (ns = not significant, * = significant *p* < 0.05, and *** = significant *p* < 0.001).

Using repeated-measures ANOVA analysis between groups showed significant differences at the various temperatures when fed on date palm pollen and immature stages of *T. urticae* for female developmental stages, reproductive phases, and fecundity; the life cycle (F = 12.260; df = 2; *p* < 0.001) (F = 14.904; df = 2; *p* < 0.001); longevity (F = 224.117; df = 2; *p* < 0.001) (F = 327.088; df = 2; *p* < 0.001); life span (F = 222.786; df = 2; *p* < 0.001) (F = 262.969; df = 2; *p* < 0.001) (Figure 1); total eggs/female (F = 22.675; df = 2; *p* < 0.001) (F = 6.500; df = 2; *p* < 0.001), and daily rate/female (F = 223.347; df = 2; *p* < 0.001) (F = 73.513; df = 2; *p* < 0.001) for *N. cucumeris* fed on date palm pollen and immature stages of *T. urticae*, respectively. A similar analysis for males also revealed significant differences for life cycle (F = 3.537; df = 2; *p* < 0.05) (F = 12.131; df = 2; *p* < 0.001), longevity (F = 186.871; df = 2; *p* < 0.001) (F = 611.789; df = 2; *p* < 0.001), and life span (F = 264.355; df = 2; *p* < 0.001) (F = 1022.879; df = 2; *p* < 0.001) (Figure 1).

### 3.2. Effect of Feeding

**Figure 2:**
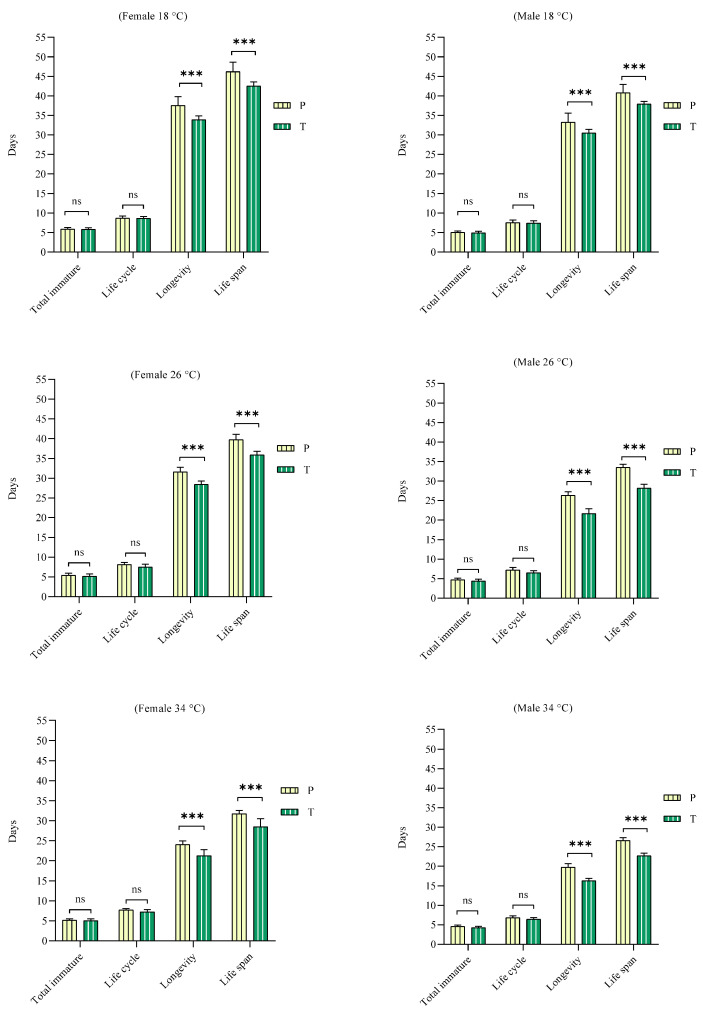
Effect of feeding (P = date palm pollen, T = *Tetranychus urticae*) on the total immature stage, life cycle, longevity, and life span period (female and male) of *N. cucumeris* (ns = not significant, and *** = significant *p* < 0.001).

The predator was able to complete its life span with both types of food at all tested temperatures. A comparison of the two types of food for *N. cucumeris* at the same temperature showed the following results from the independent-sample t-test during the life cycle, longevity, and life span. The life cycle at 18 °C (F = 0.123; df = 21.000; *p* > 0.05) (F = 0.002; df = 18.293; *p* > 0.05), 26 °C (F = 4.304; df = 17.243; *p* > 0.05) (F = 0.380; df = 17.392; *p* > 0.05), and 34 °C (F = 4.920; df = 15.970; *p* > 0.05) (F = 0.401; df = 18.969; *p* > 0.05) for females and males, respectively, demonstrated no significant difference. We observed longevity at 18 °C (F = 5.90; df = 15.188; *p* < 0.001) (F = 15.728; df = 11.448; *p* < 0.001), 26 °C (F = 0.799; df = 20.021; *p* < 0.001) (F = 1.315; df = 18.358; *p* < 0.001), and 34 °C (F = 2.705; df = 16.014; *p* < 0.001) (F = 0.761; df = 15.991; *p* < 0.001). The life span at 18 °C (F = 5.163; df = 15.662; *p* < 0.001) (F = 17.218; df = 10.595; *p* < 0.001), 26 °C (F = 4.104; df = 19.252; *p* < 0.001) (F = 2.258; df = 18.781; *p* < 0.001), and 34 °C (F = 8.439; df = 13.103; *p* < 0.001) (F = 0.58; df = 18.403; *p* < 0.001) for females and males, respectively, exhibited significant difference (Figure 2).

The generation period, that is, the period from egg to first egg laid by a female, was affected by temperature and type of food. The most prolonged generation period was 11.13 ± 0.24 days at 18 °C followed by 9.96 ± 0.22 and 9.25 ± 0.12 days at 26 and 34 °C when fed on date palm pollen. In comparison, it was 10.68 ± 0.18 days at 18 °C followed by 9.23 ± 0.24 and 8.68 ± 0.23 days at 26 and 34 °C when fed on immature stages of *T. urticae*. This difference was statistically significant (*p* < 0.05) for both types of feeding. The oviposition periods at the three different temperatures and two types of food showed significant differences (*p* < 0.05). It was longer at 18 °C 29.67 ± 0.45 and 28.27 ± 0.19 days than that observed at 26 and 34 °C when predators fed on date palm pollen and immature stages of *T. urticae*, respectively. The total number of eggs/females was significantly higher when fed date palm pollen at all temperatures than when fed *T. urticae* (Table 3 and Table 4).

The mean daily egg production per female was the highest when fed date palm pollen at 34 °C (1.95), 26 °C (1.50), and 18 °C (1.13) eggs/female/day, compared to feeding on immature stages of *T. urticae* at 34 °C (1.36), 26 °C (1.10), and 18 °C (0.82) eggs/female/day, with significant differences ((F = 5.449, df = 14.364, *p* < 0.001), (F = 0.381; df = 12.538; *p* < 0.001), and (F = 3.011; df = 15.805; *p* < 0.001)) at 18, 26, and 34 °C, respectively (Table 3 and Table 4).

**Figure 3:**
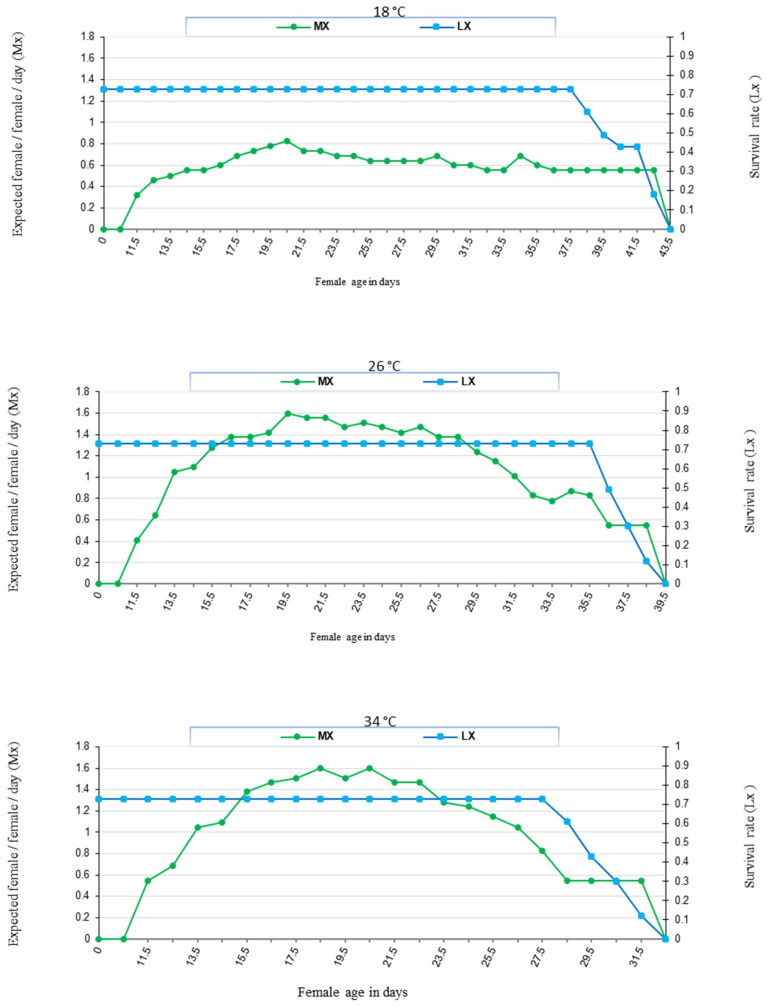
Natality and survivorship of *N. cucumeris* fed date palm pollen at (18, 26, and 34) ± 1 °C and 60 ± 5% R.H.

**Figure 4:**
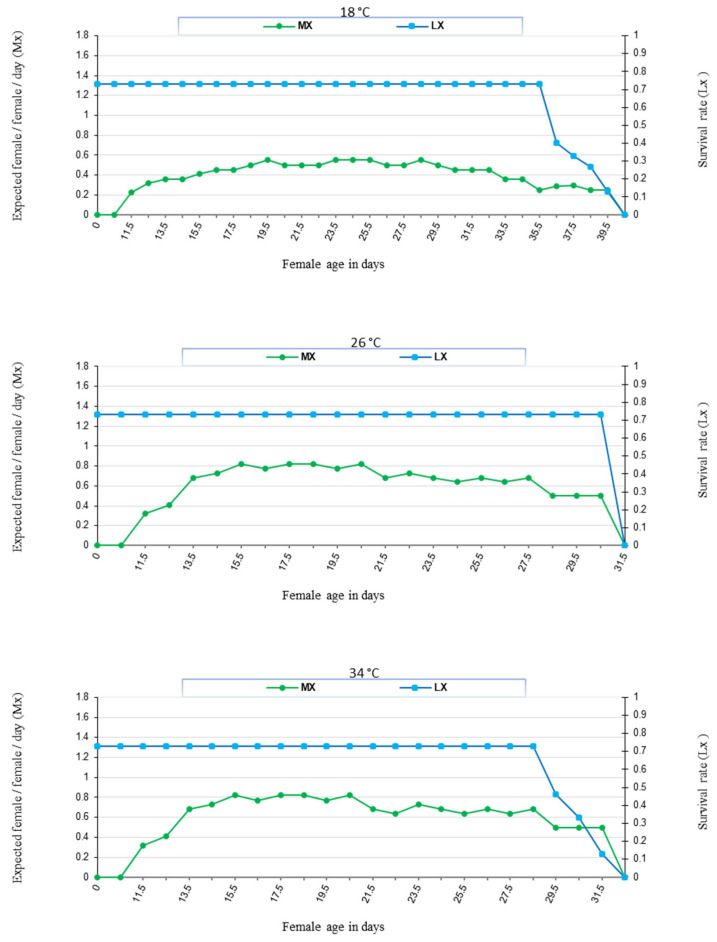
Natality and survivorship of *N. cucumeris* fed on immature stages of *T. urticae* at (18, 26, and 34) ± 1 °C and 60 ± 5% R.H.

**Figure 5:**
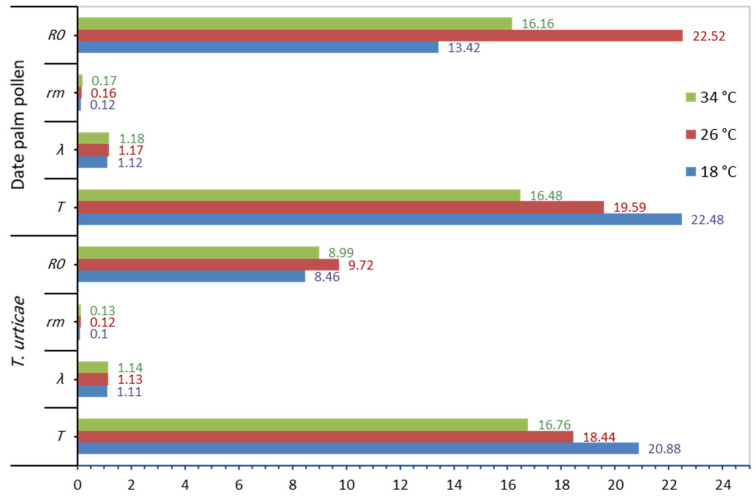
The life parameters of *N. cucumeris* fed on date palm pollen and immature stages of *T. urticae* at (18, 26, and 34) ± 1 °C and 60 ± 5% R.H.

The age-specific fecundity (Mx), rate of survival (Lx) of *N. cucumeris*, and deposited eggs were affected by the temperature and food provided (Figure 3 and Figure 4). In addition, the life table parameters (*R*_0_, *r_m_*, *λ*, and *T*) were affected by the food type at the same temperature. The data showed that *N. cucumeris*, when fed on date palm pollen, exhibited prolonged net reproductive rates (*R*_0_) of 13.42, 22.52, and 16.16 offspring/individual as compared to those fed on immature stages of *T. urticae* of 8.46, 9.72, and 8.99 offspring/individual at 18, 26, and 34 °C, respectively. The predicted number of new females who would contribute to the population daily, as indicated by the finite rate of rise (*λ*), had comparable outcomes. The finite rate of increase was influenced by the type of food used. The values of (*λ*) were the highest when the predator consumed date palm pollen at rates of 1.12, 1.17, and 1.18 day^−1^. Conversely, the rates of (*λ*) decreased when the predator fed on immature *T. urticae*, reaching the lowest rates of 1.11, 1.13, and 1.14 day^−1^ at temperatures of 18, 26, and 34 °C, respectively. The present data showed that the intrinsic rate of increase (*r_m_*) was 0.12, 0.16, and 0.17 day^−1^ when fed on date palm pollen, and the correspondent values were 0.10, 0.12, and 0.13 day^−1^ when fed on immature stages of *T. urticae* at 18, 26, and 34 °C, respectively. The mean generation time (*T*) was 22.48, 19.59, and 16.48 days when fed on date palm pollen, while that of the immature stages of *T. urticae* were the shortest at 20.88, 18.44, and 16.76 days at 18, 26, and 34 °C, respectively (Figure 5).

## 4. Discussion

Understanding how temperature and food type affect predator demographics is crucial for their application in biological control. Previous studies [19,22,41,42,43,44,45,46] have examined the effects of feeding on various types of prey, different plant pollen, and an artificial diet on the longevity and life parameters of *N. cucumeris*. This study contributes to the knowledge and provides essential data on the impact of different temperatures and food type on the growth and reproduction of the predatory mite *N. cucumeris*.

Developmental time, fecundity, mortality, reproductive period, and longevity are frequently used to assess the appropriateness of alternate and supplemental food sources for large-scale predator breeding [47,48]. In this study, *N. cucumeris* successfully developed from the egg to the adult stage on two different diets at three different temperatures. There were notable variations in the life characteristics between the two diets. Feeding date palm pollen leads to long longevity and high fecundity. Similarly, the net reproductive rate of *N. cucumeris* was greater when nourished with date palm pollen than *T. urticae* at all temperatures, exhibiting a notable disparity in reproductive rates between the two experimental diets. This might be attributed to the higher availability of immotile food sources to the predator, thereby enabling energy conservation for the remainder of the predator’s life span [24].

Several investigations have proven that a particular type of prey influences the life parameters (*R*_0_, *T*, *r_m_*) of phytoseiid mites [49,50,51,52,53]. The date pollen was compatible with large-scale cultivation of *N. barkeri* [54]. Based on the developmental period of *N. cucumeris*, it seems that pollen serves as an optimal or nearly equivalent source of food compared to tetranychid, acarid, or thrips prey species [55,56]. Researchers have reported variances in adult longevity and life parameters for *N. cucumeris* fed on different prey [57] or supplied with different pollen [58,59]. The capacity of *N. cucumeris* to consume and gain advantages from different food sources can be attributed to differences in the morphology of their mouthparts, sensory organ physiology, feeding preferences, gastrointestinal system, and behaviour [60]. Furthermore, variations in the nutritional status of the prey, along with the option between feeding on motile or immotile prey, may require distinct capacities to interpret chemical signals and engage in hunting [61]. While it is preferable to rear phytoseiid mites using their usual natural prey, this approach incurs significant labour expenses because of the requirement of obtaining fresh plants for rearing the prey [62,63]. Biological control research has recognised that the limited availability of flowering plants in simplified agricultural systems can greatly impede the survival and reproduction of parasitoids and predators. Therefore, pollen supply can greatly contribute to supporting and boosting the population of predators in the field [64]. Yazdanpanah et al. [24] confirmed that *N. cucumeris* reared on pollen diets had higher quality than those reared on natural prey *T. urticae*; therefore, date palm pollens are good candidates for the mass rearing of *N. cucumeris* for use in augmentative biological control programs.

Our findings revealed that this predator can undergo development and reproduction throughout a broad temperature range from 18 to 34 °C. Temperature also affected survival rates, fecundity, and survival rate and influenced the net reproductive rate value.

Female and male longevity was the longest (37.54 and 33.25 days) at 18 °C and the shortest (24.04 and 19.75 days) at 34 °C, respectively, when fed date palm pollen. Similarly, when feeding on *T. urticae* immature stages, the longevity of females and males was the longest (33.91 and 30.50 days) at 18 °C and the shortest (21.27 and 16.27) days at 34 °C, respectively. Our results also revealed that the mean duration of the egg and all immature stages decreased with increasing temperature, with no significant differences. Similar results have been reported by Mohamed et al. [65], who found that the female and male longevity of *N. cucumeris* when fed on movable stages of *Panonychus ulmi* (Koch) at 20 °C was longest than 25 and 30 °C. Similar trends have been reported for three other *Neoseiulus* species, *N. longispinosus* [66], *N. barkeri* [51], and *N. neoagrestis* [67], who reported that pre-oviposition, post-oviposition period, and female longevity decreased as temperature increased.

The average total number of eggs laid per female was the highest at 26 °C (40.17) when they fed on date palm pollen and (26.64) when they fed on immature stages of *T. urticae*, followed by 34 °C, which was higher than that determined at 18 °C. Mohamed et al. [65] reported that *N. cucumeris* females deposited an average of 23.6, 24.0, and 18.0 eggs at 20, 25, and 30 °C, respectively, when fed on movable stages of *P. ulmi*. Ji et al. [68] found that *N. cucumeris* females deposited an average of 53.3 eggs when fed on *Carpoglyphus lactis* under 25 °C. *Neoseiulus californicus* fed on *T. urticae* produced 41.6, 38.4, and 28.4 eggs at 20, 25, and 30 °C, respectively [69]. Bonde [49] reported that the total number of eggs laid by *N. barkeri* females fed on *Thrips tabaci* was 47.1 eggs at 25 °C. These results clearly show that the effects of temperature on egg production by phytoseiid mites vary depending on the species [67].

Our results revealed that the highest net reproductive rate (*R*_0_) was 22.52 offspring/individual when they were fed date palm pollen and 9.72 offspring/individual when they were fed *T. urticae* at 26 °C. Yazdanpanah et al. [24] reported that (*R*_0_) of *N. cucumeris* fed on *T. urticae* at 25 ± 1 °C was 18.39, 6.15, 12.34, 8.71 for generation one (G1), (G10), (G20), and (G30), respectively. Moradi et al. [67] reported that the highest (*R*_0_) *of N. neoagrestis* fed on *Tyrophagus putrescentiae* was determined at 25 °C compared to 20 °C and 30 °C.

In addition, our results demonstrated that the highest intrinsic rate of natural increase (*r_m_*) was (0.17 day^−1^) when they fed on date palm pollen and (0.13 day^−1^) when they fed on *T. urticae* immature stages at 34 °C. Furthermore, the highest finite rate of increase (*λ* = 1.18 day^−1^) was observed when fed on date palm pollen and (1.14 day^−1^) when fed on immature stages of *T. urticae* at 34 °C. It seems that the shorter life span of females is the primary factor for increasing (*r_m_*). The primary influence of temperature on the (*r_m_*) value of *N. cucumeris* was primarily due to its impact on the predator’s developmental time. Sabelis and Janssen [70] reported that variations in the developmental rate had a greater impact on the (*r_m_*) than predatory mite oviposition rates. The smaller (*r_m_*) value at 18 °C may be attributed to reduced fecundity and survival rate, limited female offspring production in the population, and an extended overall life span. *Neoseiulus cucumeris* reached its maximum (*r_m_*) value at 34 °C in the present study, indicating that it is a thermophilic species, despite its highest survival and fecundity values at 26 °C. El Taj and Jung [71] reported that for *N. californicus* fed on *P. ulmi*, (*R*_0_) was highest at 25 °C, and both (*r_m_*) and (*λ*) were highest at 30 °C. Comparatively, researchers found that the thermophilic species *Euseius finlandicus* thrives at a temperature of 30 °C [72]. The intrinsic rate of increase (*r_m_*) of *Amblyseius californicus* [73], *Euseius scutalis* [74], and *Neoseiulus barkeri* [75] increased as the temperature increased from 20 to 30 °C. Nevertheless, a proficient predator must possess an inherent rate that is at least equivalent to that of its prey to effectively decrease its population [76].

The present study determined that the ideal temperature for promoting population growth of *N. cucumeris* under a date palm pollen and *T. urticae* diet was 26 °C. In our study, we concluded that *N. cucumeris* was successfully fed date palm pollen as an alternative food source, completing its developmental stages and recording the highest net reproduction rate at all tested temperatures when compared with *T. urticae*. Therefore, the inflorescences of date palm trees contain a significant quantity of pollen, potentially reducing labour costs, and these pollens can maintain their quality as a food source for several months. As a result, date palm pollen provides a simple, efficient, readily available, and easily stored food source for *N. cucumeris*, possibly enhancing its value compared to alternative options in field settings. In addition, the immature stages of *T. urticae* are suitable as food sources for *N. cucumeris* because they shorten the mean generation time. Implementing alternative diets for mass rearing may effectively reduce the expenses associated with long-term mass rearing and sustain the effectiveness of biocontrol measures.

## Figures and Tables

**Table 1:** Duration in days (mean ± S.E.M) of the developmental stages of *N. cucumeris* fed date palm pollen at (18, 26, and 34) ± 1 °C and 60 ± 5% R.H.

Stage	Sex		Temperature	
		18 °C	26 °C	34 °C
Egg	♀	2.79 ± 0.07 ^a^	2.71 ± 0.07 ^a^	2.54 ± 0.10 ^a^
	♂	2.55 ± 0.09 ^a^	2.50 ± 0.11 ^a^	2.30 ± 0.08 ^a^
Larva	♀	1.25 ± 0.08 ^a^	1.21 ± 0.07 ^a^	1.17 ± 0.07 ^a^
	♂	1.20 ± 0.08 ^a^	1.15 ± 0.08 ^a^	1.10 ± 0.07 ^a^
Protonymph	♀	1.88 ± 0.07 ^a^	1.79 ± 0.07 ^a^	1.71 ± 0.07 ^a^
	♂	1.55 ± 0.09 ^a^	1.40 ± 0.07 ^a^	1.35 ± 0.08 ^a^
Deutonymph	♀	2.75 ± 0.08 ^a^	2.42 ± 0.10 ^a^	2.29 ± 0.07 ^a^
	♂	2.25 ± 0.08 ^a^	2.15 ± 0.08 ^a^	2.10 ± 0.07 ^a^
Life cycle	♀	8.67 ± 0.15 ^a^	8.13 ± 0.15 ^ab^	7.71 ± 0.10 ^b^
	♂	7.55 ± 0.20 ^a^	7.20 ± 0.21 ^a^	6.85 ± 0.13 ^a^
Longevity	♀	37.54 ± 0.65 ^a^	31.58 ± 0.34 ^b^	24.04 ± 0.26 ^c^
	♂	33.25 ± 0.75 ^a^	26.30 ± 0.29 ^b^	19.75 ± 0.29 ^c^
Life span	♀	46.21 ± 0.70 ^a^	39.71 ± 0.40 ^b^	31.75 ± 0.24 ^c^
	♂	40.80 ± 0.67 ^a^	33.50 ± 0.26 ^b^	26.60 ± 0.23 ^c^

Using one-way ANOVA and the LSD post hoc test, the means in rows followed by different letters are significantly different (*p* < 0.05).

**Table 2:** Duration in days (mean ± S.E.M) of the developmental stages of *N. cucumeris* fed on immature stages of *T. urticae* at (18, 26, and 34) ± 1 °C and 60 ± 5% R.H.

Stage	Sex	Temperature		
		18 °C	26 °C	34 °C
Egg	♀	2.77 ± 0.08 ^a^	2.27 ± 0.08 ^a^	2.18 ± 0.08 ^a^
	♂	2.50 ± 0.07 ^a^	2.14 ± 0.07 ^a^	2.14 ± 0.07 ^a^
Larva	♀	1.23 ± 0.08 ^a^	1.18 ± 0.08 ^a^	1.14 ± 0.07 ^a^
	♂	1.18 ± 0.08 ^a^	1.09 ± 0.06 ^a^	1.09 ± 0.06 ^a^
Protonymph	♀	1.86 ± 0.07 ^a^	1.73 ± 0.08 ^a^	1.64 ± 0.07 ^a^
	♂	1.50 ± 0.10 ^a^	1.18 ± 0.10 ^a^	1.14 ± 0.07 ^a^
Deutonymph	♀	2.73 ± 0.08 ^a^	2.27 ± 0.08 ^a^	2.27 ± 0.08 ^a^
	♂	2.23 ± 0.08 ^a^	2.09 ± 0.06 ^a^	2.05 ± 0.05 ^a^
Life cycle	♀	8.59 ± 0.15 ^a^	7.45 ± 0.24 ^b^	7.23 ± 0.17 ^b^
	♂	7.41 ± 0.18 ^a^	6.50 ± 0.17 ^b^	6.55 ± 0.13 ^b^
Longevity	♀	33.91 ± 0.29 ^a^	28.41 ± 0.26 ^b^	21.27 ± 0.46 ^c^
	♂	30.50 ± 0.28 ^a^	21.68 ± 0.37 ^b^	16.27 ± 0.19 ^c^
Life span	♀	42.50 ± 0.33 ^a^	35.86 ± 0.28 ^b^	28.50 ± 0.61 ^c^
	♂	37.91 ± 0.20 ^a^	28.18 ± 0.30 ^b^	22.68 ± 0.21 ^c^

Using one-way ANOVA and the LSD post hoc test, the means in rows followed by different letters are significantly different (*p* < 0.05).

**Table 3:** Adult female longevity and fecundity (mean ± S.E.M in days) of *N. cucumeris* fed date palm pollen at (18, 26, and 34) ± 1 °C and 60 ± 5% R.H.

°C	Generation Period	Pre-Oviposition	Oviposition	Post-Oviposition	No. of Eggs/Female	
					Total Average	Daily Rate
18	11.13 ± 0.24 ^a^	2.46 ± 0.13 ^a^	29.67 ± 0.45 ^a^	5.42 ± 0.29 ^a^	33.42 ± 1.03 ^a^	1.13 ± 0.03 ^a^
26	9.96 ± 0.22 ^b^	1.83 ± 0.09 ^b^	26.83 ± 0.21 ^b^	2.92 ± 0.19 ^b^	40.17 ± 0.58 ^b^	1.50 ± 0.02 ^b^
34	9.25 ± 0.12 ^c^	1.54 ± 0.11 ^b^	20.08 ± 0.23 ^c^	2.42 ± 0.15 ^b^	39.08 ± 0.58 ^c^	1.95 ± 0.03 ^c^

Using one-way ANOVA and the LSD post hoc test, the means in a column followed by different letters are significantly different (*p* < 0.05).

**Table 4:** Adult female longevity and fecundity (mean ± S.E.M in days) of *N. cucumeris* fed on immature stages of *T. urticae* at (18, 26, and 34) ± 1 °C and 60 ± 5% R.H.

°C	Generation Period	Pre-Oviposition	Oviposition	Post-Oviposition	No. of Eggs/Female	
					Total Average	Daily Rate
18	10.68 ± 0.18 ^a^	2.09 ± 0.06 ^a^	28.27 ± 0.19 ^a^	3.55 ± 0.16 ^a^	23.18 ± 0.40 ^a^	0.82 ± 0.01 ^a^
26	9.23 ± 0.24 ^b^	1.77 ± 0.08 ^b^	24.18 ± 0.23 ^b^	2.45 ± 0.16 ^b^	26.64 ± 0.54 ^b^	1.10 ± 0.02 ^b^
34	8.68 ± 0.23 ^c^	1.45 ± 0.11 ^b^	18.09 ± 0.31 ^c^	1.73 ± 0.19 ^c^	24.64 ± 0.97 ^c^	1.36 ± 0.05 ^b^

Using one-way ANOVA and the LSD post hoc test, the means in a column followed by different letters are significantly different (*p* < 0.05).

## Data Availability

The original contributions presented in this study are included in the article. Further inquiries can be directed to the corresponding author.
